# Nursing students’ clinical judgment skills in simulation and clinical placement: a comparison of student self-assessment and evaluator assessment

**DOI:** 10.1186/s12912-023-01220-0

**Published:** 2023-03-09

**Authors:** Anne Mette Høegh-Larsen, Marianne Thorsen Gonzalez, Inger Åse Reierson, Sissel Iren Eikeland Husebø, Dag Hofoss, Monika Ravik

**Affiliations:** 1 Department of Nursing and Health Sciences, Faculty of Health and Social Sciences, University of South-Eastern Norway, Postbox 235, Kongsberg, 3603 Norway; 2 Department of Quality and Health Technology, Faculty of Health Sciences, University of Stavanger, Stavanger, Norway; 3 Department of Surgery, Stavanger University Hospital, Stavanger, Norway

**Keywords:** Nursing education research, Nursing students, Clinical judgment, Simulation training, Clinical placement, Self-assessment

## Abstract

**Background:**

Clinical judgment is an important and desirable learning outcome in nursing education. Students must be able to self-assess their clinical judgment in both the simulation and clinical settings to identify knowledge gaps and further improve and develop their skills. Further investigation is needed to determine the optimal conditions for and reliability of this self-assessment.

**Aims:**

This study aimed to compare the same group of students’ self-assessment of clinical judgment with an evaluator’s assessment in both simulation and clinical settings. The study further aimed to investigate whether the Dunning-Kruger effect is present in nursing students’ self-assessment of clinical judgment.

**Methods:**

The study applied a quantitative comparative design. It was conducted in two learning settings: an academic simulation-based education course, and a clinical placement course in an acute care hospital. The sample consisted of 23 nursing students. The Lasater Clinical Judgment Rubric was used to collect data. The scores were compared using a *t*-test, intraclass correlation coefficient, Pearson’s correlation coefficient, and Bland-Altman plots. The Dunning-Kruger effect was investigated using linear regression analysis and a scatter plot.

**Results:**

The results showed an inconsistency between student self-assessment and evaluator assessment of clinical judgment in both simulation-based education and clinical placement. Students overestimated their clinical judgment when compared to the more experienced evaluator’s assessment. Differences between students’ scores and the evaluator’s scores were larger when the evaluator’s scores were low, indicating the presence of the Dunning-Kruger effect.

**Conclusion:**

It is vital to acknowledge that student self-assessment alone may not be a reliable predictor of a student’s clinical judgment. Students who had a lower level of clinical judgment were likely to be less aware that this was the case. For future practice and research, we recommend a combination of student self-assessment and evaluator assessment to provide a more realistic view of students’ clinical judgment skills.

## Background

Clinical judgment skills are required to provide safe patient care and is therefore an important and desired learning outcome in nursing education [1–4]. The term clinical judgment skills are defined by Benner and Tanner [5] (p200) as “the ways in which nurses come to understand the problems, issues, or concerns of clients and patients, to attend to salient information, and to respond in concerned and involved ways”. Simulation-based education and clinical placement are the learning activities in nurse education most relevant to facilitating the development of students’ clinical judgment [2, 3, 6–9]. Failure for students to receive educational support and thereby achieve an adequate level of clinical judgment constitutes a major threat to patient safety, potentially leading to negative consequences for patients and society [10, 11].

Assessment of student competence is a pillar of education and is necessary to determine students’ further learning needs [12–14]. Hence, it is important to assess nursing students’ level of clinical judgment in the simulation setting and the clinical setting. Having the most accurate picture possible of nursing students’ clinical judgment skills can help educators identify knowledge gaps that hinder students in making sound clinical judgments [13]. By identifying students’ knowledge and skill gaps, educators can further support the development of competence to better meet professional nursing care standards for patients with multifaceted issues [6, 8, 13, 14].

Nursing students’ clinical judgment skills can be assessed by an evaluator, such as a faculty member or clinical supervisor, or by students themselves using self-assessment [8, 11, 15]. Evaluators who perform assessments must be trained in observing and mapping more objectively based on observations, as well as in the use of the instrument assessing the skills in question [16]. Andrade [17] defines self-assessment as “the act of monitoring one’s processes and products in order to make adjustments that deepen learning and enhance performance”. As used in education, self-assessment is considered to promote students’ responsibility for and self-regulation of their own learning [18]. Students’ ability to judge the quality of their own and other’s work is vital for patient safety and healthcare quality [19]. This capability can also be defined as evaluate judgment and such skill might support students’ learning after graduating [19, 20]. Self-regulatory skills such as self-assessment and evaluating judgment may support students in directing and regulating their actions towards learning outcomes and are thus necessary for the transition from novice student to lifelong learner in clinical practice [12, 18, 19, 21]. In research, self-assessment is commonly used to explore and describe students’ behaviour, skills, performance, and experiences [16]. Additionally, student self-assessment is often chosen in education and research to minimize the resources required, such as faculty and researcher staff time [16–18, 22].

Students’ self-assessment processes have been investigated in various ways. Consistency between different assessment methods has been found to be valuable for identifying students’ knowledge gaps and subsequently improving their nursing skills, performance, and behaviour [16]. Consistency has typically been investigated by comparing students’ self-assessments with an experienced evaluator’s assessment [21, 23]. To our knowledge, three previous studies [24–26] have compared nursing students’ self-assessment and evaluators’ assessment of students’ clinical judgment using the Lasater Clinical Judgment Rubric (LCJR) [6]. The use of rubrics is considered the key to reliable assessment in education and research [27]. Rubrics include assessment criteria, levels of performance and the weights of each criterion [28]. According to a recent systematic literature review, LCJR is currently the most recognized instrument for assessing nursing students’ clinical judgment [29]. Two of the three previous studies comparing students’ and evaluator assessment were conducted in the simulation setting [24, 26] while the third study [25] was conducted in the clinical setting. The overall conclusion in all these three studies is that students tend to overestimate their clinical judgment skills in both the simulation and the clinical setting when compared to an evaluator assessment. However, none of the studies investigated the same students in different settings, even though it has been argued that self-assessment should be investigated under different settings [16, 17, 21, 23]. Thus, looking at the same group of students’ self-assessment of clinical judgment skills in two settings may provide valuable knowledge.

Addressing the process of assessing clinical judgment skills, the response bias from students’ self-assessment is of interest as it may act as a barrier to reflection and learning [16]. One example of response bias is the Dunning-Kruger effect [30], which identifies that individual with low competence overestimate their competence. The Dunning-Kruger effect can be identified by a simple calculation of the difference between a student’s subjective self-assessment and a more objective assessment performed by an experienced evaluator [16, 30]. If the Dunning-Kruger effect is present among nursing students and they are unable to recognize their deficits in clinical judgment, relying heavily on student self-assessment of clinical judgment may lead to inaccurate evaluations in educational learning outcomes and research, and ultimately threaten patient safety and patient care [16, 31, 32].

Irrespective of the benefits mentioned above and the established use of self-assessment of nursing students’ clinical judgment skills in education and research, knowledge gaps concerning the assessment process still exist. As the organizational and pedagogical approaches used in the simulation setting and the clinical setting differ, it is of interest to investigate the self-assessment process in both settings. Such knowledge may enable educators to apply appropriate pedagogical approaches to further develop students’ clinical judgment. To our knowledge, there are no existing studies comparing the same group of students’ self-assessments with evaluators’ assessments in two different settings. Moreover, no previous studies have investigated whether the Dunning-Kruger effect [30] is present in nursing students’ self-assessment of clinical judgment skills.

Thus, this study aimed to compare the same students’ self-assessments of clinical judgment with evaluators’ assessments in both simulation and clinical settings. The study further aimed to investigate whether the Dunning-Kruger effect [30] is present in nursing students’ self-assessment of clinical judgment. The research questions were as follows:

1. Did nursing students’ self-assessment of clinical judgment in the simulation setting reflect their clinical judgment as assessed by an evaluator?
2. Did the same nursing students’ self-assessment of clinical judgment in the clinical placement setting reflect their clinical judgment as assessed by an evaluator?
3. Is the Dunning-Kruger effect present in nursing students’ self-assessment of clinical judgment in the simulation setting or the clinical setting?

## Methods

### Research design

This study uses a quantitative, comparative design and is reported in accordance with the STROBE guidelines (Additional file 1) and the Reporting Guidelines for Health Care Simulation Research [33]. The study is part of a larger study addressing nursing students’ professional competence and clinical judgment.

### Research settings

The study took place in the second year of a three-year Bachelor of Nursing programme at a Norwegian university. This undergraduate nursing education programme entailed 180 credits in the European Credit Transfer and Accumulation System (ECTS) [34]. More specifically, the nursing students got 90 ECTS credits from theoretical courses mainly in the academic setting, minimum 75 ECTS credits from clinical placement in a variety of settings, and maximum 15 ECTS credits from simulation-based education in laboratories [34]. The study was conducted in two learning settings: a simulation centre on the university campus and an acute care hospital unit.

In the simulation setting, the students took part in a two-day simulation-based education course comprising six simulation sessions focusing on different deteriorated patient conditions and diagnoses. Nine faculty members were involved as facilitators and operators. Students were divided into groups of six to nine, alternating between the roles of nurse and observer. The simulation environment mirrored a patient room in a hospital unit and Laerdal SimMan 3G™ and ALS™ manikins were used. Each simulation session (90 min) consisted of a prebriefing (15 min), a simulated scenario (15 min), a viewing of the video recording of the simulated scenario (15 min), and a facilitator-led group debriefing (45 min). For the debriefing, the Promoting Excellence and Reflection in Simulation (PEARLS) structured and scripted debriefing [35] method was used.

After the simulation-based education course, the students attended an eight-week clinical placement course in a medical or surgical hospital unit hosting adult patients with acute, critical, and chronic conditions. Students provided nursing care under the supervision of a registered nurse working in the relevant unit. Nurse educators supervised the students in groups to promote reflection and learning and to evaluate their learning outcomes.

The learning outcomes for both courses entailed the same clinical judgment skills.

### Recruitment and participants

The target group for the study was second-year nursing students. In advance of the recruitment, all students had completed theoretical courses addressing pathology and core nursing issues related to patients in need of acute care, had passed a six-week clinical placement course in a nursing home, were certified in cardiopulmonary resuscitation, and had attended compulsory classes in practical nursing skills. For recruiting, information about the study was published on the university’s digital learning platform and distributed in a pre-clinical course by the first author. Eligible participants were informed about the study aim, data collection methods, confidentiality, voluntary participation, and their right to withdraw. A sample size calculation showed that 16 student-evaluator comparisons were sufficient to identify an average 2-point difference between student and evaluator scores on the LCJR, with a standard deviation of 4 points. Due to the predetermined organisation of the simulation-based education and the clinical placement courses, it was impossible to collect data from more than one student at a time. Consequently, the study allowed for a maximum of 24 participants out of the 89 students attending the courses. Of these, the first 24 students who signed up to participate were formally invited, of which N = 23 participated. The sample consisted of 19 women (82.6%) and four men (17.4%), with ages ranging from 20 to 54 years (Mean = 28 years). None of the participants had previous experience with scenario simulation, and 78.3% had experience working in healthcare services.

### Measure

The Norwegian version of the Lasater Clinical Judgment Rubric (LCJR-N) [9] was used to collect data concerning nursing students’ clinical judgment skills. The original LCJR was developed by Lasater [6] to directly observe and evaluate students’ individual performance of clinical judgment in a simulation setting. It was designed to provide a common language for learners, faculty, and preceptors to talk about learners’ thinking and to serve as a help for offering formative guidance and feedback [6, 11]. It is based on Benner’s novice to expert model [36] and Tanner’s clinical judgment model [4]. LCJR has emerged as a tool used by evaluators for observation and by students for self-assessment in both simulation and clinical settings [11, 15, 37]. The LCJR corresponded well to students’ learning outcomes in the simulation-based education course and the clinical placement course. The LCJR consists of four dimensions, called subscales in the present study, with a total of 11 items: *Noticing* (3 items), *Interpreting* (2 items), *Responding* (4 items), and *Reflecting* (2 items) [6]. The items on students’ performance were scored from 1 to 4 with higher scores indicating better clinical judgment: 1 point = beginning, 2 points = developing, 3 points = accomplished, and 4 points = exemplary [6]. The total score ranges from 11 to 44.

The LCJR has been translated into Norwegian, Swedish, German, Chinese and several other languages [9, 25, 38, 39]. In a recent review, internal consistency was supported for both evaluator and student self-assessment [15]. Regarding reliability and validity for the Norwegian version of LCJR (LCJR-N) in previous research, Cronbach’s alphas (0.74–0.91) indicated good internal consistency and face validity was verified [9]. In the current study, the Cronbach’s alphas for the LCJR-N total score ranged from 0.87 to 0.91, and from 0.69 to 0.85 for the *Noticing* and *Responding* subscales. Alpha values were not calculated for the *Interpreting* and *Reflection* subscales as these scales only had two items each.

### Data collection

Data were collected from students and the evaluator in December 2019 in the simulation setting and in February 2020 in the clinical setting. Data on students’ self-assessments using the LCJR-N were collected by self-reported questionnaires in pen and paper format together with demographic information. Data from the evaluator were also collected by using LCJR-N in pen and paper format.

In the simulation setting, the evaluator completed the LCJR-N for each student while observing the student in the simulation scenario. Data for the subscale *Reflecting* was collected by observing the students in the debriefing. Immediately after the simulation-based education course, the LCJR-N was handed out to the students. Each student completed the LCJR-N while recalling the simulation scenario, in which they had monitored vital signs on the manikin. The questionnaires were distributed and collected by faculty members who were not otherwise engaged in the study.

In the clinical setting, the same evaluator completed the LCJR-N for each student while observing the students in a patient care situation where the student monitored a patient’s vital signs. Data for the subscale *Reflecting* were collected by posing three questions to each student after they left the patient’s room (“If you had to do it again, would you do anything differently?”, “What would you do then?”, and “Why would you do this differently?”). Immediately after, each student completed the LCJR-N while recalling the patient care situation. The LCJR-Ns were distributed to the students and collected by the evaluator.

The term “evaluator” in this study refers to first author, who is a registered nurse (RN) with a Master’s degree in Nursing Science (MSN), a researcher, and a faculty member. The evaluator was not involved in any of the participants’ educational activities. The evaluator has years of experience with the simulation setting and pedagogical approaches in simulation-based education, as well as with supervising and assessing students in clinical placements. Moreover, the evaluator is a clinically experienced RN having worked 15 years in acute care units entailing using clinical judgment skills when caring for deteriorating patients. The evaluator’s preparatory work for data collection included examining the concept of clinical judgment and using LCJR-N as an observation tool by testing it in a simulation scenario. During this preparation, the evaluator corresponded with the LCJR’s developer Kathie Lasater regarding the use of the LCJR in various assessments and the use of only one evaluator. Because using only one evaluator may create evaluation biases [40], this issue was carefully considered. The credibility of data collected by only one evaluator was considered acceptable and in line with reported findings in a recent review by Lee [15] demonstrating high interrater reliability metrics for the LCJR. All students had previous experience with use of the LCJR-N from having participated in an earlier research study.

### Statistical analysis

Data were analysed by SPSS version 28.0. A paired-samples *t*-test was used to compare the students’ and the evaluator’s LCJR-N scores. Intraclass correlation coefficient (ICC) was used to investigate degrees of correlation and agreement between students’ and the evaluator’s LCJR-N scores, in line with the suggestions of Koo and Li [41]. ICC estimates and their 95% confidence intervals were based on a mean rating (*k* = 2), consistency, and a 2-way mixed-effects model [41]. ICC was interpreted in line with Landis and Koch [42], with values ≤ 0.20 indicating slight agreement, from 0.21 to 0.40 indicating fair agreement, 0.41 to 0.60 indicating moderate agreement, 0.61 to 0.80 indicating substantial agreement, and ≥ 0.81 indicating almost perfect agreement. Pearson’s correlation coefficient was used to investigate the relationship between students’ and the evaluator’s LCJR-N scores. Pearson’s correlation coefficients were interpreted as *r* = 0.10, 0.30, and 0.50 indicating a small, medium, or large correlation, respectively [43]. Bland-Altman plots were created to illustrate the average bias and to investigate whether there were systematic differences between students’ and the evaluator’s LCJR-N scores [44].

Linear regression analysis was used to investigate whether the Dunning-Kruger effect was present in nursing students’ self-assessment of clinical judgment skills in the simulation setting or the clinical placement setting. The linear regression analysis determined whether the discrepancy between student LCJR-N scores and evaluator LCJR-N scores was the same across the evaluator’s LCJR-N scores or increased with lower values on the evaluator’s LCJR-N scores. A scatter plot was created to illustrate the results of the linear regression.

The *p*-value for statistical significance was set at < 0.05.

## Results

### Comparison of student self-assessment and evaluator assessment of students’ clinical judgment in the simulation setting

**Fig. 1:**
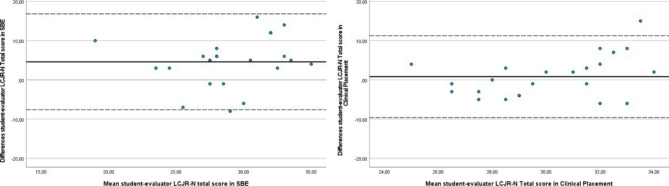
Bland-Altman plots of students’ self-assessment and evaluator’s assessment on LCJR-N total scores in simulation-based education (SBE) and clinical placement

In the simulation setting, students’ LCJR-N total score and subscale scores were significantly higher than the evaluator’s scores (Table 1). The Pearson’s correlation coefficients for student and evaluator assessments for both total score and subscales were quite low (-0.01 to 0.32), with none of them reaching statistical significance (Table 2). The ICC of the LCJR-N total score and the subscale *Noticing* ranged from − 0.01 to 0.17, indicating “slight agreement” between the students’ and the evaluator’s assessments. The ICC scores for the subscales *Interpreting*, *Responding*, and *Reflecting* ranged from 0.32 to 0.39, indicating “fair agreement” between the students’ and evaluator’s scores in the simulation setting (Table 2). The Bland-Altman plots showed a systematic difference and wide limits of agreement between students’ and evaluator’s LCJR-N total score and subscale scores. The Bland-Altman plots for all LCJR-N subscales and total score illustrated that students’ scores were higher than the evaluator’s score. Figure 1 shows an example of the Bland-Altman plot for the LCJR-N total score in the simulation setting.

**Table 1:** Comparison of students’ self-assessment and evaluator’s assessment on LCJR-N

	In the simulation setting			In the clinical setting		
	Students	Evaluator		Students	Evaluator	
Variables	Mean ± SD	Mean ± SD	*p*	Mean ± SD	Mean ± SD	*p*
LCJR-N						
Total score	31.26 ± 5.28	26.65 ± 4.37	0.002*	30.48 ± 4.40	29.65 ± 2.81	0.465
Noticing	8.52 ± 1.76	6.39 ± 1.47	0.000*	8.04 ± 1.43	7.65 ± 0.83	0.274
Interpreting	5.39 ± 1.20	4.65 ± 1.11	0.038*	5.35 ± 1.03	5.35 ± 0.78	1.000
Responding	11.09 ± 1.93	10.00 ± 1.83	0.027*	10.91 ± 1.89	10.61 ± 1.08	0.475
Reflecting	6.26 ± 1.18	5.61 ± 1.12	0.029*	6.17 ± 0.89	6.04 ± 1.02	0.613

Note. LCJR-N = Lasater Clinical Judgment Rubric Norwegian Version. *p < 0.05

**Table 2:** Agreement and correlation between students’ self-assessment and evaluator’s assessment on LCJR-N

	In the simulation setting				In the clinical setting			
Variables	ICC	95% CI	*p*	*r*	ICC	95% CI	*p*	*r*
**LCJR-N**								
Total score	0.17	-0.25 - 0.54	0.210	0.18	-0.04	-0.44 - 0.37	0.581	-0.05
Noticing	-0.01	-0.41 - 0.39	0.522	-0.01	-0.02	-0.42 - 0.39	0.540	-0.03
Interpreting	0.39	-0.37 - 0.44	0.429	0.04	-0.26	-0.60 - 0.16	0.892	-0.27
Responding	0.32	-0.10 - 0.64	0.063	0.32	0.14	-0.28 - 0.52	0.257	0.16
Reflecting	0.32	-0.09 - 0.64	0.062	0.32	0.19	-0.23 - 0.55	0.187	0.19

Note. LCRJ-N = Lasater Clinical Judgment Rubric Norwegian Version; ICC = Intraclass Correlation Coefficient

### Comparison of student self-assessment and evaluator assessment of students’ clinical judgment in the clinical setting

In the clinical setting, students’ LCJR-N total score and subscale scores were higher than the evaluator’s scores; however, this difference was not significant (Table 1). The Pearson’s correlation coefficient (*r*) for student and evaluator assessments on LCJR-N total score and subscales were quite low (-0.27 to 0.19) and none of them reached statistical significance (Table 2). The ICC values of the LCJR-N total score and all subscales ranged from − 0.26 to 0.19, indicating “slight agreement” between the students’ and the evaluator’s assessments (Table 2). The Bland-Altman plots indicated a systematic difference and wide limits of agreement between students’ and the evaluator’s LCJR-N total score and all subscale scores. Each Bland-Altman plot showed that students’ scores were higher than the evaluator’s scores. Figure 1 shows an example of the Bland-Altman plot for LCJR-N total score in the clinical setting.

### The Dunning-Kruger effect in students’ self-assessment of clinical judgment

**Fig. 2:**
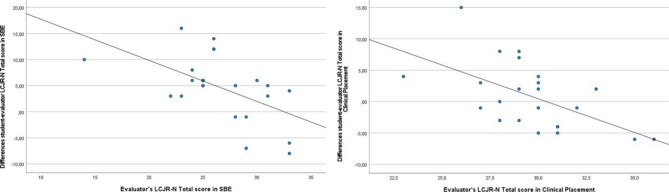
Scatter plots of differences in student-evaluator LCJR-N total scores by evaluator’s LCJR-N total score in simulation-based education (SBE) and clinical placement

In the simulation setting, the linear regression analysis of LCJR-N total score and subscales showed that the difference between the students’ scores and the evaluator’s score increased significantly as the evaluator’s score decreased (Table 3; Fig. 2). This means that the differences between student and evaluator scores were larger when the evaluator’s score was low.

**Table 3:** Changes in student-evaluator differences on LCJR-N by evaluators assessment on LCJR-N

	In the simulation setting		In the clinical setting			
Variables	b	*p*	b	*p*		
LCJR-N						
Total score Student-Evaluator difference	-0.787	0.006*	-1.076	0.005*		
Noticing Student-Evaluator difference	-1.015	< 0.001*	-1.043	0.011*		
Interpreting Student-Evaluator difference	-0.958	< 0.001*	-1.362	< 0.001*		
Responding Student-Evaluator difference	-0.662	0.006*	-0.717	0.071		
Reflecting Student-Evaluator difference	-0.660	0.006*	-0.833	< 0.001*		

Note. LCJR-N = Lasater Clinical Judgment Rubric Norwegian Version. *p < 0.05

In the clinical setting, the linear regression analysis of LCJR-N total score and the subscales *Noticing*, *Interpreting*, and *Reflecting* showed that the difference between student and evaluator scores increased significantly as the evaluator’s score decreased (Table 3; Fig. 2). In absolute terms, the patterns were similar for the subscale *Responding*, however, this regression effect was not significant (Table 3; Fig. 2).

## Discussion

### Comparison of student self-assessment and evaluator assessment of students’ clinical judgment

Comparing students’ self-assessment and evaluator assessment of students’ clinical judgment skills, the overall results showed an inconsistency in both the simulation and clinical settings. Students assessed their clinical judgment as being higher than the evaluator did. When comparing the assessments using* t*-tests, this difference was significant in the simulation setting but not in the clinical setting. However, using supplementary statistical tests such as Pearson’s r, ICC, and scatter plots, we found the inconsistency between student and evaluator assessment to be present independently of the learning setting. These findings regarding assessment of nursing students’ clinical judgment both in the simulation and clinical settings add valuable knowledge to this research field.

Because no existing research has investigated the same group of students in two educational settings, in what follows we compare our results with findings from research conducted in one educational setting. The student-evaluator inconsistency identified in our study concurs with previous studies concluding that students tend to overestimate their clinical judgment compared to evaluators [24–26]. In accordance with our findings from the simulation setting, Strickland and Cheshire [26] found student self-assessment in the simulation setting to be higher than evaluator assessment, and they reported a positive, although not strong, correlation (*r* = 0.31) between these assessments. Likewise, Jensen [24] found that students rated themselves higher than the evaluator did in the simulation setting, although not significantly higher. Corresponding to our findings, Jensen [24] also reported weak correlations (*r* = -0.14–0.27) between students’ assessment and evaluators’ assessment. In accordance with our findings from the clinical placement setting, Vreugdenhil and Spek [25] found the student-evaluator difference to be systematic and significant (*p* = 0.020) when investigating agreement, with students tending to score themselves significantly higher (6.1%) than the evaluator did. As in our findings, Vreugdenhil and Spek [23] did not find any significant differences between students’ and evaluators’ assessments in a *t*-test analysis, but they did find a strong positive correlation (*r* = 0.78) between students’ self-assessment and evaluator assessment, which is different from our findings. Taken together, previous studies and our study show that students tend to rate their clinical judgment higher than the evaluator, regardless of being studied in the simulation or the clinical setting and regardless of being studied in one or two settings.

The inconsistency between the same students’ and same evaluator’s assessments in the simulation setting and the clinical setting in our study may have several explanations. The student-evaluator inconsistency might be due to different understandings of the concept of clinical judgment. Although students were trained in the use of the LCJR-N, the items on which corresponded to the learning outcomes in the simulation-based education course and the clinical placement course, their cognitive or linguistic representation of clinical judgment might still be limited [45]. On this issue, the use of a rubric as LCJR-N in the assessment process could make it easier for students and evaluators to recognize the expectations for clinical judgment [6]. However, assessing clinical judgment is complicated and requires metacognitive skills, the ability to think abstractly, and an in-depth understanding of nurses’ responsibilities and role in the clinical setting [29]. The students and the evaluator might have had different perceptions of clinical judgment and therefore interpreted the assessment criteria in the LCJR-N differently. The students might have focused on specific tasks more than on cognitive processes in clinical judgment. Novice students often lack capability to reflect abstractly on theoretical and practical aspects of a skill and thus tend to focus on superficial features of their performance in the self-assessment process [16, 23]. This argument aligns well with Benner’s “from novice to expert” theory [36], which identifies five levels of competence in nursing – novice, advanced beginner, competent, proficient, and expert – each of which builds upon the previous one. Benner [36] describes nursing students as being at a novice level, characterised by having little experience and understanding of the situations in which they are expected to perform, which is often the case. In the current study, students’ limited experience might have affected their understanding of the contextual meaning of clinical judgment and how to apply these skills in simulation scenarios or clinical situations, and thus also influenced their self-assessments.

### The Dunning-Kruger effect in student self-assessment of clinical judgment

To investigate whether the Dunning-Kruger effect was present in students’ self-assessment of clinical judgment, the evaluator’s score for students’ clinical judgment was considered more accurate due to the evaluator’s training in the use of LCJR-N and her higher level of education, competence, and experience.

The findings indicate that the Dunning-Kruger effect was present in students’ self-assessment of clinical judgment in both the simulation setting and the clinical setting due to the larger student-evaluator differences when the evaluator’s score was low. These findings are consistent with findings reported in a recent review on the Dunning-Kruger effect in a variety of educational contexts in the social sciences [16].

An explanation for our findings might be students’ lack of metacognitive awareness of their own clinical judgment. In other words, the students who were assessed by evaluator as having a lower level of clinical judgment were unaware of having a low level, and therefore were more likely to overestimate their clinical judgment [30]. It is questionable whether novice nursing students’ halfway through a bachelor’s programme have sufficient metacognitive skills and a sufficient level of self-reflection to accurately assess their own clinical judgment. Metacognitive skills involving assessing one’s own competence develop through self-evaluation, self-reflection, and feedback from others [16, 30]. Reflection itself is vital and valued in simulation-based education and clinical placement in nursing education. Reflection in these settings comprises students’ assessment of their actions and previous clinical situations followed by the integration of new knowledge and adjustment of clinical performance [4, 46]. Such assessment might promote learning and has the potential to develop students’ evaluative judgment and further lifelong learning [19, 47]. In the simulation setting in this study, students’ self-reflection on clinical judgment was carefully promoted by using the student-centred and structured Promoting Excellence and Reflection in Simulation (PEARLS) debriefing [35]. While student-centred and structured debriefing approaches have the potential to provide students with optimal opportunities for reflection and increased activity [48, 49], a pitfall in debriefing is that facilitators do not appropriately close all relevant performance gaps [50]. Hence, the facilitator might not have been attentive to students’ performance gaps concerning clinical judgment, resulting in missed learning opportunities.

Acknowledging the potential for the Dunning-Kruger effect in students’ self-assessment of clinical judgment in nursing education offers opportunities for establishing meaningful feedback discussions while learning and improving [16]. As novice students gradually develop metacognitive skills over the course of their education, blind spots regarding their own clinical judgment might decrease [36]. Hence, students’ metacognitive skills and the potential presence of the Dunning-Kruger effect should always be considered when deciding on an assessment method for nursing student’s clinical judgment.

### Limitations

The study has some limitations. The sample size and the fact that there was only one sampling site limit the generalisability of the findings. There may also be measurement errors due to the use of only one evaluator [51]. Despite the evaluator’s theoretical and practical preparation to avoid observational biases, having only one evaluator eliminated the possibility of doing interrater reliability analysis on scores between evaluators [40]. Although the evaluator was prepared for the observation and not involved with students from other learning activities, objective observation and assessment of skills such as clinical judgment is always a challenge [40, 51]. Another potential measurement error is that students’ behaviour in the data collection situations might have been atypical due to their awareness of being observed [52], also known as the Hawthorne effect [53]. Finally, there is also a risk of instrumentation bias as the LCJR-N has not been psychometrically tested for the Norwegian context.

### Implications for education and future research

Although nursing students’ self-assessment is widely used and considered valuable for evaluation and learning [15, 16, 29], our findings urge caution when interpreting nursing students’ self-assessment of clinical judgment in education. Students’ ability to determine their own level of competence and identify knowledge gaps is decisive for clinical performance within the limits of their competence in a lifelong learning perspective [19, 54–56]. Therefore, nurse educators should facilitate students’ metacognitive skills and their evaluative judgement related to clinical judgment. Further, acknowledging the presence of the Dunning-Kruger effect among nursing students may inspire faculty to promote students’ metacognitive skills and self-reflection, thereby supporting students in their learning process [16]. Promoting nursing students’ self-reflection regarding clinical judgment by using LCJR in simulation-based education and various clinical placement settings may help students gain a deeper understanding of the concept of clinical judgment before graduating. For future educational assessment practice in simulation and clinical settings, a combination of assessment methods is recommended [13]. Student self-assessment, evaluator assessment, and feedback may offer a more realistic interpretation of students’ clinical judgment and help faculty to identify those students who require additional support during their education before graduation [24, 26, 57].

For future research, pedagogical interventions aiming to promote nursing students’ metacognitive skills in relation to clinical judgment using controlled designs should be performed. Researchers should be aware of the Dunning-Kruger effect and its potential impact on validity when having students’ self-assessments as the only data source. Moreover, the Dunning-Kruger effect among nursing students should also be investigated using larger samples and other instruments. Finally, studies using a qualitative approach to explore nursing students’ experiences from self-assessment of clinical judgment are welcomed.

## Conclusion

This study contributes to the body of knowledge regarding assessment of nursing students’ clinical judgment using the LCJR-N in the field of nursing education and research. Overall, our findings indicate an inconsistency between student self-assessment and evaluator assessment in the simulation setting and in the clinical setting, with students tending to have a higher estimation of their own clinical judgment compared to an evaluator’s assessment. The findings further demonstrate that the Dunning-Kruger effect was present in our sample, as students whom the evaluator assessed as having a lower level of clinical judgment were likely to be unaware of their own low level.

For future practice and research, it is vital to acknowledge that student self-assessment alone may not be a reliable predictor of a student’s clinical judgment. Thus, we recommend a combination of student self-assessment and evaluator assessment to provide a more realistic view of students’ clinical judgment.

## Data Availability

The datasets used and/or analysed during the current study are available from the corresponding author on reasonable request.
